# The performance and environmental impact of pro-oxidant additive containing plastics in the open unmanaged environment—a review of the evidence

**DOI:** 10.1098/rsos.230089

**Published:** 2023-05-10

**Authors:** Fabiola Sciscione, Helen C. Hailes, Mark Miodownik

**Affiliations:** ^1^ UCL Plastic Waste Innovation Hub, University College London, London, UK; ^2^ Mechanical Engineering Department, University College London, London, UK; ^3^ Department of Chemistry, University College London, 20 Gordon Street, London, UK

**Keywords:** oxo-degradable, oxo-biodegradable, biodegradable, pro-degradant, pro-oxidants, microplastics

## Abstract

Pro-oxidant additive containing (PAC) plastics is a term that describes a growing number of plastics which are designed to degrade in the unmanaged natural environment (open-air, soil, aquatic) through oxidation and other processes. It is a category that includes ‘oxo-degradable’ plastics, ‘oxo-biodegradable’ plastics and those containing ‘biotransformation’ additives. There is evidence that a new standard PAS 9017 : 2020 is relevant to predicting the timescale for abiotic degradation of PAC plastic in hot dry climates under ideal conditions (data reviewed for South of France and Florida). There are no reliable data to date to show that the PAS 9017 : 2020 predicts the timescale for abiotic degradation of PAC plastics in cool or wet climatic regions such as the UK or under less ideal conditions (soil burial, surface soiling etc.). Most PAC plastics studied in the literature showed biodegradability values in the range 5–60% and would not pass the criteria for biodegradability set in the new PAS 9017 : 2020. Possible formation of microplastics and cross-linking have been highlighted both by field studies and laboratory studies. Systematic eco-toxicity studies are needed to assess the possible effect of PAC additives and microplastics on the environment and biological organisms.

## 1. Introduction

Polyolefins are the largest class of commodity thermoplastic polymers. They include polymers of alkene hydrocarbons such as polypropylene (PP), polyethylene (PE), polystyrene (PS) and high, low and linear-low density polyethylene (HDPE, LDPE and LLDPE), and vinyl polymers such as polyvinyl chloride (PVC). They account for 75% of the global plastic production. The durability and resistance to degradation of these plastics are due to their high molecular weight, hydrophobicity and absence of functional groups, which make them resistant to attack by microbial enzymes, light, water etc. [1]. The same properties that have made plastics so widespread and useful to humans have also created serious environmental problems. Globally, 22% of the annual plastic production enters terrestrial and aquatic environments where they can remain for decades [2].

**Figure 1.**
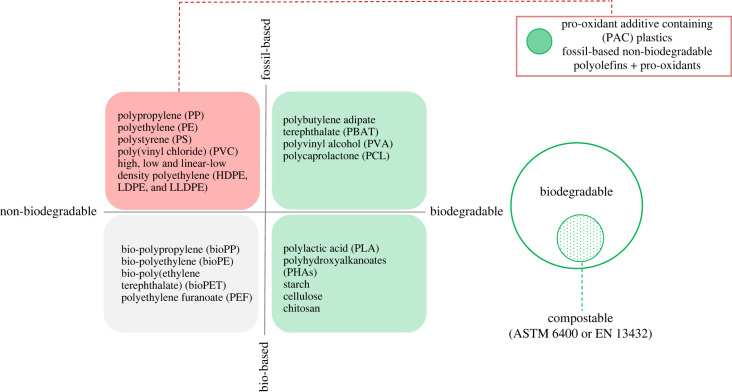
Schematic showing the different families of plastics.

One strategy to reduce plastic pollution has been to design polyolefin-based materials to degrade more quickly in the air under UV light and heat. The degradation process can be accelerated by the addition of pro-oxidants (also referred to as pro-degradants). The most used pro-oxidants are transition metals (e.g. Fe, Co, Mn, Cu, Ce or Ni) in the form of salts (e.g. carboxylates, dithiodicarbamates, acetylacetonates) or organic complexes. They are usually added to the polyolefin blend up to a 5% loading by weight. Pro-oxidants based on transition metal ions with organic ligands have been developed by several companies and are sold as master batches. Transition metals can act as catalysts in the polyolefin photo- and thermal-degradation processes. These processes are triggered by UV light and/or heat and oxygen and proceed via radical chain reactions which result in the production of low-molecular weight oxidation products (oxygen is introduced into the carbon chain in the form of hydroxyls, carbonyls and peroxides). Polyunsaturated compounds have also been used to introduce ‘weak points’ in the polymer chain during polymerization to accelerate the rate of degradation. Eventually, the polymer degrades into smaller fragments of low molecular weight that are assumed to be assimilated by microorganisms. Fossil-based polyolefins containing pro-oxidant additives, and more recently as ‘biotransformation’ additives, are known as ‘oxo-degradable’ or ‘oxo-biodegradable’ plastics, and more recently but more generally as pro-oxidant additive containing **(**PAC) plastics. They are different in design and structure from traditional polyolefins, bio-based plastics, biodegradable and compostable plastics, as illustrated in figure 1.

For over 40 years PAC plastics have been marketed as a solution for soil and marine littering. However, concerns have been raised recently in this regard. In the 2010 research report by Loughborough University [3] commissioned by DEFRA on PAC plastics, the authors found that the time required for the degradation of PAC plastics cannot be predicted accurately, as it depends on the environmental conditions, and estimated that 2–5 years are necessary for these plastics to degrade in the open environment in the UK. Other issues raised are the contamination of composting and recycling streams by PAC plastics. In 2017 the European Commission released a report entitled ‘Study to provide information supplementing the study on the impact of “oxo-degradable plastic” on the environment’ [4]. The authors analysed 13 claims either made by the PAC industry or common-held beliefs. Some of these hypotheses were found to be supported by evidence, others partially supported, others inconclusive, and some refuted. The results highlighted the unsuitability of PAC plastics for composting, landfill and recycling; they are summarized in table 1.

**Table 1.** Summary of the key findings on PAC plastics by the EU report [5].

hypothesis	evidence
Hypothesis 1: In open environments, oxo-biodegradable additives will accelerate the fragmentation of traditional polymers	supported
Hypothesis 2: PAC plastics should not be considered compostable	supported
Hypothesis 3: In open environment, PAC plastics biodegrade following their fragmentation	partially supported
Hypothesis 4: PAC plastics do not biodegrade in landfill	supported
Hypothesis 5: PAC plastics biodegrade in marine environments	inconclusive
Hypothesis 6: In soil, fragmented and potentially partially degraded plastics and their additives pose limited negative effects to soil quality or ecosystems	inconclusive
Hypothesis 7: The use of PAC plastic does not instil or promote a throwaway attitude	inconclusive
Hypothesis 8: PAC plastic is a possible solution to reduce the problems of plastic marine litter compared with conventional plastic	inconclusive
Hypothesis 9: PAC plastics can be identified and separated in collection systems	refuted
Hypothesis 10: PAC plastics can be identified and separated within recycling processes	refuted
Hypothesis 11: The quality of conventional plastics recyclate is not negatively affected by PAC plastic added to the feedstock	refuted
Hypothesis 12: The presence of PAC plastics in recyclate does not affect recyclate prices or marketability	refuted
Hypothesis 13: The presence of PAC plastic in recyclate does not affect the ability of manufacturers to guarantee specific business requirements relating to physical properties (such as tensile strength etc.)	market dependent

Following this analysis, and because of ‘a lack of consistent evidence about the rate of abiotic and biotic decomposition in the environment’ [6], the European Union banned the use of PAC plastics from the 3 July 2021. This decision to ban a class of plastics provoked interest by other governments from around the world seeking to understand the best way to protect the environment from plastic waste. It additionally raises the question—should other governments also ban PAC plastics?

This literature review seeks to shed light on the debate by examining the evidence. We address four outstanding questions regarding PAC plastics pertaining to whether they biodegrade rapidly and completely in the environment, whether this can be predicted accurately by laboratory tests, and whether they do so causing no toxicity or other ill effects to biological organisms and the environment. The specific questions are as follows:

— Is there evidence that correlates laboratory weathering tests of PAC plastics to their behaviour in the unmanaged natural environment?
— What are the findings from real field-test studies?
— Are microplastics formed during the degradation of PAC plastics?
— Are there ecotoxicity concerns relating to the PAC plastic additives?

This paper is structured in the following way: first we provide a review the biodegradation process of PAC plastics and the standards and test methods for their biodegradability (§2), see table 2. Then we review the evidence for each question in turn (§§3–6). Finally, we discuss and synthesize the evidence for policymakers (§7).

## 2. The biodegradation of pro-oxidant additive containing plastics

Biodegradation is a complex process which generally happens in three stages [7,8]:

*Stage 1: Abiotic and biotic deterioration*.

The abiotic stage involves fragmentation of the polymer by the action of mechanical forces, photo- and/or thermal oxidation to smaller molecular-weight fractions that can be more vulnerable to the microbial action. Biodeterioration is the first step of biodegradation where the surface of the polymer is colonized by microorganisms (bacteria, fungi, yeast and algae). The growth of microbes on the polymer surface is essential for the secretion of extracellular enzymes.

*Stage 2: Bio-fragmentation*

During bio-fragmentation, extracellular enzymes break down the polymer into smaller fragments (oligomers, monomers) increasing the ‘accessibility’ of the materials to microbial action. For polymers that are inherently non-biodegradable, such as polyolefins, microbes can rely on the abiotic factors to break down the polymer into fragments small enough to enter the cell and be further cleaved by internal enzymes.

*Stage 3: Bio-assimilation (or mineralization)*

Complete biodegradation is achieved when the monomers are assimilated by microbial organisms and converted into biomass, CO_2_ and H_2_O in the presence of oxygen (aerobic conditions). Methane (CH_4_) can also be produced in the absence of oxygen (anaerobic conditions).

The rate of the abiotic degradation of polymers containing pro-oxidants is analysed in terms of their loss of mechanical properties (e.g. elongation at break, tensile strength, Young's modulus), reduction of molecular weight (including number average molecular weight (*M*_n_), weight average molecular weight (*M*_W_) and higher average molecular weight (*M*_z_)) by gel permeating chromatography (GPC) and increase of the carbonyl index (CI) by Fourier-transform infrared spectroscopy (FT-IR) or attenuated total reflectance (ATR)-FT-IR. Other complementary techniques are contact angle (CA) to monitor the increase of the surface hydrophilicity, and differential scanning calorimetry (DSC) to determine the thermal properties and particularly the degree of crystallinity of the polymer under study. The latter can also be studied in parallel with other techniques such as X-ray diffraction (XRD).

The rate of biotic degradation can be determined by several methods, each one presenting some limitations [8,9]. For this reason, more than one method is usually used to assess biodegradability. The main methods used are summarized in table 3. Other techniques such as surface hydrolysis measurements, gas chromatography–mass spectrometry (GC-MS), liquid chromatography–mass spectrometry (LC-MS), gel permeation chromatography (GPC), nuclear magnetic resonance (NMR) and Fourier transform spectroscopy (FT-IR) have also been employed as complementary methods.

**Table 2.** Summary of pass/fail criteria specified by the PAS9017:2020.

	experimental condition	pass criteria
**weathering of polyolefins**		
film samples		
	accelerated weathering machine equipped with UV light.	CI ≥ 1
	irradiance: 0.8 W m^−2^ (±0.02 W m^−2^)	molecular weight change:
	UV cycle time: 1 h	*M*_n_ < 5000 Da
	dark cycle time: 23 h	*M*_z_ < 30 000 Da
	air chamber temperature: 60°C (±0.02°C)	*M*_w_ loss > 90%
timeframe	no more than 14 days	within 14 days
standards methods	ASTM D4329 or BS EN ISO 4892-1	for CI
	ASTM D6474 or BS ISO 1614-4	for molecular weight determination
laboratory-outdoor exposure correlation	*ca* 3 months of outdoor exposure under South Florida weather conditions.	
rigid samples		
	accelerated weathering machine equipped with xenon-arc light	CI ≥ 1
	irradiance: 0.35 W m^−2^ (±0.02 W m^−2^) with 340 nm daylight filter	molecular weight change:
	UV cycle time: 8 h	*M*_n_ < 5000 Da
	dark cycle time: 16 h	*M*_z_ < 30 000 Da
	air chamber temperature: 60°C (±2°C)	*M*_w_ loss >90%
	uninsulated black panel temperature: 70°C (±2°C)	
timeframe	no more than 28 days	within 28 days
standards methods	ASTM D52565 or BS EN ISO 4892-2	for CI
	ASTM D6474 or BS ISO 1614-4	for molecular weight determination
laboratory-outdoor exposure correlation	*ca* four months of outdoor exposure under South Florida weather conditions.	
ecotoxicity testing		
pre-weathering		
test	OECD 202: *Daphnia* sp., acute immobilization test and reproduction test	no more than 10% of daphnids immobilized in the control.
		dissolved oxygen concentration ≥3 mg l^−1^ (control and test vessel).
post-weathering		
test	OECD 208: Terrestrial plants: seedling emergence and seedling growth test	seedling emergence ≥ 70%
	OECD 211: *Daphnia magna* reproduction test	no visible phytotoxic effects
	OECD 222: Earthworm reproduction test	mean survival of emerged control seedling ≥ 90%
		mortality of female *Daphnia* ≤ 30%
		mean number of offspring > 60%
		dissolved oxygen concentration ≥2 mg l^−1^ (control and test vessel).
		≥ 30 juveniles per replicate (10 adults)
		30% coefficient of variation of reproduction
		10% adult mortality within four weeks
biodegradation of wax after weathering		
to be performed only on polyolefin materials that have passed the criteria for degradation specified in the weathering section above		
	natural or standard soil	≥ 90% CO_2_ conversion
	mesophilic conditions	
timeframe	within 2 years	
test method	ASTM D5988 or BS EN ISO 17556	

The best practice to study the biodegradation of PAC plastics is to expose the plastic material to natural weathering conditions and monitor the abiotic and biotic degradation in the real environment. However, the time required to achieve complete biodegradation can take years and also be limited by cost factors. For this reason, accelerated laboratory tests are normally preferred to facilitate the introduction of a new material onto the market. For this purpose, accelerated UV laboratory weathering tests, with a fluorescent-UV or xenon-arc test chamber, are used to simulate the performance (e.g. durability) that a polymer material would have in the actual environment of use under long-term weathering. Usually, the accelerated laboratory conditions specified in standard test methods, in terms of duration and intensity of UV light, temperature, humidity, angle of exposure etc., are representative of specific geographical locations such as Florida, Arizona or South of France. According to ASTM D6954 [10] and test method BS EN ISO 4892-1 [11], the results obtained from the laboratory accelerated weathering and outdoor exposure should be used to compare performances between different plastic materials in a particular environment and should not be representative of the performances of those materials in a different environment. Likewise, it is not possible to extrapolate absolute results on the rate of degradation of different materials in the environment as these will depend on the type of material and its specific physico-chemical properties (e.g. polymer chain length, molecular weight, crystallinity, porosity, number of tertiary carbons and functional groups etc.).

In controlled environments, such as composting facilities or anaerobic digesters, the biodegradation of a plastic material can be determined under standardized procedures allowing reproducibility across different laboratory studies. The situation is different in the unmanaged natural environment where the rate of degradation will depend on several environmental factors such as the climate of the geographic position and the season, both of which will influence the amount of UV light, temperature and humidity experienced by a plastic material. Also, different ecosystems, both natural (soil, freshwater, marine) and man-made (landfill), will have different environmental factors affecting the rate of biodegradation (e.g. pH, temperature, moisture content etc.). Biodegradation can also be affected by different factors within the same environment. For example, the rate of degradation will be different for a material floating on the surface of the sea, experiencing higher temperature, oxygen and UV light, compared with in deep waters where the photo- and thermal oxidation might be limited due to reduced amounts of oxygen and light, and lower temperatures. The same consideration may be applied to materials located above- or in-soil, and in landfills, which are dark and anaerobic environments. Ultimately, in the uncontrolled environment, mixtures of different types and concentrations of e.g. bacteria, fungi or yeast are present which further complicate the standardization and reproducibility of these studies in a controlled laboratory setting.

### 2.1. Standards for biodegradation of pro-oxidant additive containing plastics

Standards can be divided into two categories: test methods and specifications. The former defines procedure and protocols that need to be followed (e.g. how to carry out a test/measurement). The latter specifies a series of pass/fail criteria that need to be met for a product to be compliant with the standard, for example to be classified as biodegradable or compostable. Alongside specification standards, certification labels for compostable and biodegradable plastics are used to provide clear information to customers on the conformity of the product to defined pass/fail criteria from accepted standards.

The most frequently used standards to confirm the biodegradability of PAC plastics are ASTM D6954 [10] and BS 8472 [12]. They are based on a three-tier system divided into:

(1) Tier 1—Abiotic degradation: laboratory accelerated tests using UV light or heat in the presence of oxygen to promote oxidation and degradation of the polymer.
(2) Tier 2—Biotic degradation: the abiotically degraded polymer is further broken down by the action of microorganisms.
(3) Tier 3—Ecotoxicity: the final product of the abiotic and biotic degradation is tested for its toxicity on plants and earthworms.

These standards do not set clear pass criteria and cannot be considered as specification standards. Other standards which specify pass criteria are the Swedish SPCR 141 [13], which is based on ASTM 6954 and BS 8472, and the French AFNOR AC T51 808 [14]. A detailed table detailing the standards can be found in the Eunomia report [4].

In 2020 a new publicly available specification PAS 9017 [15] was developed to provide methods, timescales and pass criteria for demonstrating that polyolefins containing biodegradable additives will biodegrade in the open-air terrestrial environment (i.e. littering or unmanaged disposal) without forming microplastics. The PAS is divided into three parts:

(1) Weathering of polyolefins: film or rigid polyolefin samples are subjected to laboratory accelerated weathering under specific conditions (light, air, heat) and timeframes specified in the PAS which correspond to a specific time period of outdoor exposure under South Florida conditions. The rate of degradation is tested by CI and molecular weight analysis. These two are the keys parameters specified in the PAS 9017 to determine whether a PAC plastic can break down and meet the requirement for soil biodegradability. The CI gives an indication of the extent of oxidation of polyolefins as a result of the weathering process. CI is measured by attenuated total reflection Fourier Transform infrared spectroscopy (ATR-FT-IR) by monitoring the absorption band of the carbonyl species formed during photo- or thermo-oxidation processes in the range of 1850–1650 cm^−1^ and comparing its area with the area under the carbonyl peak relative to a reference standard. This value should be greater than 1.0 after artificial or outdoor weathering, as specified in PAS 9017, for a film or rigid sample to be qualified for soil-biodegradability. Molecular weight changes are expressed in terms of number average molecular weight (*M*_n_), weight average molecular weight (*M*_w_) and higher average molecular weight (*M*_z_) and measured by GPC. These values must be *M*_n_ < 5000 Da, *M*_z_ < 30 000 Da and *M*_w__loss_ > 90% according to the PAS 9017. A wax is expected to form as a result of the weathering process.
(2) Ecotoxicity testing: on potential biologically hazardous substances present on the surface or in the polymer matrix.
(3) Biodegradation of the wax after weathering: in mesophilic conditions using soil as the medium for the test. A respirometry test is used to measure the amount of organic carbon in the wax converted to CO_2_ or O_2_ consumed.

Experimental conditions and pass criteria are specified in the PAS 9017 documentation [15]. The soil biodegradation test, included in the PAS 9017 : 2020, is BS EN ISO 17556 [5] which only covers laboratory tests and does not apply to open-air environment tests.

## 3. Is there evidence that correlates laboratory weathering tests of pro-oxidant additive containing plastics to their behaviour in the unmanaged natural environment?

### 3.1. Mechanisms of pro-oxidant additive containing plastics degradation

The abiotic and biotic degradation of PAC plastics in the unmanaged natural environment has been mainly assessed under controlled laboratory conditions simulating real-life scenarios. A summary of the key publications can be found in table 4. Evidence that PAC plastics can physically degrade into lower molecular weight fragments upon exposure to light and heat has been demonstrated and the oxidative mechanisms are now well understood and accepted [4,6] [16–20]. Several papers have studied the biodegradation of PAC plastic by microorganisms following fragmentation [19–27]. In these studies, pro-oxidant containing polyolefin films were previously aged by photo- and/or thermal oxidation before incubation with microorganisms. In one study by Jakubowicz [23] a level of biodegradation of 91% was achieved for LDPE films containing Mn stearate after a 753-day incubation period in soil. In all other cases a broad range of biodegradation was observed from 5% to 60% (table 4). The discrepancies observed in the levels of biodegradability reflect the differences in the experimental conditions (light intensity, temperature, exposure time), nature and concentration of the pro-oxidant, and chemical structure of the polyolefin. For example, Fontanella *et al.* [24] studied three matrices of HDPE, LDPE, LLDPE with or without three different pro-oxidant additives: P1 complex (Mn + Fe), P2 complex (Mn + Fe) and P3 complex (Mn + Fe + Co) with stearate ligands. Cobalt containing P3 complex induced the highest degree of oxidation (highest molecular weight reduction) in all the three matrices examined, with the HDPE matrix being the least sensitive to oxidation in the presence of every pro-oxidant additive. Nevertheless, upon incubation with *Rhodococcus rhodochrous* strain in the mineral medium, matrixes containing P3 complex showed toxicity against *R. rhodochrous* due to the high concentration of cobalt stearate. Mineralization levels for LDPE and LLDPE film containing P1 complex were also determined in soil and were 9% and 12%, respectively. HDPE film containing P3 pro-oxidant showed a mineralization lower than 5%. These results might be explained by the different structure of the examined polyolefins. The higher degree of branching and higher number of tertiary carbons of LDPE makes it less resistant to oxidation compared with HDPE. The branching also prevents PE chains from stacking close together and allows LDPE chains and tertiary carbons to be more accessible to microbial attack.

**Table 3.** Main methods for determining biodegradability of plastics.

method	use	limitations
scanning electron microscopy (SEM)	useful for visualizing microbial growth, cracks and pits on the surface of the polymer	the presence of microbes on the surface does not provide evidence of complete biodegradation
mass loss (gravimetric analysis)	weight loss resulting from microbial consumption of low molar masses	only useful for detecting early stage degradation as polymer recovery from culture media is not accurate and particles resulting from abiotic degradation (microparticles) may be missed
mineralization (or level of biodegradation)	respirometric methods are used in international standards to assess the complete biodegradation by the rate of conversion of the organic carbon into CO_2_ and/or CH_4_	biomass and additional components, such as inorganic additives, that are not converted into biogas are not accounted for; this can lead to inaccuracy; valid only if polymer is the only carbon source
biomass growth	to measure the biomass growth as a result of the mineralization process	only useful in highly controlled environments where the polymer is the only carbon source

**Table 4.** Recent peer-reviewed publications for the biodegradability of PAC plastics under controlled laboratory conditions.

author	reference	polyolefin (film thickness)	pro-oxidant	abiotic treatment	biotic treatment	mineralization level (%)
Chiellini 2003	[19]	LDPE film	TDPA (Epi Inc.)	thermo­oxidation 55°C, 44 days	soil (25°C) and compost (25°C), 600 days	49–60%
Chiellini 2007	[20]	LDPE films (*ca* 30 m)	10–15% wt/wt TDPA (EPI)	thermal oxidation (50–70°C)	incubation in river water, 100 days	10–40% (from extract)
Jakubowicz 2003	[22]	LDPE film (30 m)	Mn stearate (low and high concentration) (EKM)	thermal oxidation 50–70°C, 28 days	soil, 60°C, 200 days (ISO14855 and prEN 14046)	60–65%
Jakubowicz 2011	[23]	LDPE film (15 m)	Mn stearate (P- Life Japan Inc.)	thermal oxidation 40–75°C, 10 days	soil (25°C) and compost (25°C), 733 days (ISO 17556 and ISO 17556)	91%
Reddy 2009	[28]	LDPE film (60–80 µm)	n.s.	thermal oxidation 50–70°C, 14 days	*P. aeruginosa* in mineral medium, 42 days	no biodegradation; molecular weight increases after 42 days incubation
Koutny 2006	[21]	HDPE (20 µm) LDPE (60 m)	Fe photo­inducer (n.s.) + organometallic-type thermoinducer (EPI) + phenolic antioxidant	photo­-oxidation, 120 days + thermal oxidation, 60°C, 300 h	*R. rhodochrous* and *Nocardia asteroides* in mineral medium, 141–203 days	no biodegradation; only superficial deterioration by ATP/ ADP measurements
Fontanella 2010	[24]	HDPE, LDPE, LLDPE (43–59 m)	(Me + Fe) complex P1; (Me +Fe) complex P2; (Me +Fe + Co) complex P3; complex = stearate; manufacturer n.s.	photo-­oxidation, 60°C, 40h + thermal oxidation, 60°C, 300h	soil (25°C) + compost (58°C), 352 days	soil: 5–12%, compost: 16–24%
					*R. rhodochrous* strain in mineral medium for 180 days	*R. rhodococcus* (ATP/ ADP measurements only)
Fontanella 2013	[25]	random PP co­polymer	Mn, Mn + Fe, or Co stearate; manufacturer n.s.	photo-­oxidation, 60°C, 40h + thermal oxidation, 60°C, 300h	*R. rhodochrous* strain in mineral medium for 180 days	*R. rhodococcus* (ATP/ ADP measurements only)
		block-PP copolymer (51–63 µm)				
Abrusci 2011	[26]	LDPE LLDPE (25 m)	Ca or Fe stearate (0.2%wt)	photo-oxidation (300–800 nm), 550 W m−^2^; 45°C, 500 h	mix of *Bacillus*	11.5% highest value for LDPE-Ca and LDPE-Fe incubated with *B. borstelensis*
			Repsol		*Brevibacillus borstelensis*, 90 days, 30 or 45°C	
Abrusci 2013	[27]	LDPE LLDPE (25 m)	Fe, Co, Mn stearate (0.2%wt) Repsol	photo-oxidation (300–800 nm), 550 W m^−2^; 45–70°C, 500 h, 9 days	mix of *Bacillus*	59.2% highest values for LDPE-Co incubated with *B. borstelensis*
					*Brevibacillus borstelensis*, 90 days, 45°C	
Eyheraguibel 2017	[29]	HDPE blown films (20 µm) Ribeyron SA	Fe photoinducer; orgonametallic-Mn thermo inducer + phenolic antioxidants	photo-oxidation s, 60°C + thermal ­oxidation, 60°C, 300 h	*R. rhodochrous*, 240 days	n.d. biodegradation assessed by consumption of oligomers by
					biodegradation on oligomers extracted after abiotic ageing	*B. rhochrous* (ATP/ ADP; NMR and LC-MS)
Rose 2020	[30]	LDPE and oxo- LDPE films (1% dg12–08 symphony Env. Ltd)	n.s.	photo-oxidation	*R. rhodochrous*, *Alcanivorax borkumensis*	CO_2_ evolved by microorganisms (gas chromatograophy); no levels of absolute biodegradability reported
					incubation 35 days	

The degree of crystallinity seems to play a crucial role in determining the extent of oxidation in PAC plastics. This is because oxygen diffusion and microbial attack are more likely to happen at the amorphous regions of the polymer where impurities (carbonyls, double bonds etc.) are also present and can function as ‘anchors’ for the microbial attack [8,16,25,27,31,32]. The role of the polyolefin chemical structure and concentration and type of pro-oxidant were also studied by Abrusci *et al.* [26]. Similarly to the studies of Fontanella *et al.*, they found the pro-oxidant effect increased in the order Fe-stearate < Mn-stearate < Co-stearate after abiotic treatment (photo or thermal) of pro-oxidant containing LDPE films. However, in this case, in contrast to Fontanella *et al.*, the presence of much lower concentrations of Co-stearate did not show any toxicity and instead resulted in the highest mineralization level upon incubation with either a mixture of three bacterial strains or *B. borstelensis*.

Some authors have assessed the biodegradability of PAC plastics by measuring the increase in the CI, decrease in molecular weight and mechanical properties and in the formation of a biofilm on the surface of the polymer. Generally, a reduction in molecular weight of oxo-PE films was observed in most studies after abiotic treatment [19,22,24,26–28,33]. However, as recently pointed out by Montazer *et al.* [34], these reports failed to distinguish between partial and complete biodegradation. There is no current consensus that characterization techniques of abiotic and biotic deterioration stage can predict complete biodegradation. For instance Reddy and co-workers [28] pre-aged oxo-PE samples which showed a decrease in molecular weight and increase in CI as a result of the abiotic degradation. Upon incubation with *Pseudomonas aeruginosa* the formation of a biofilm on the surface was observed for the oxo-PE film samples. To understand whether the extent of biodegradation was only limited to the surface of the polymer, molecular weights were analysed during the six-week incubation period with *P. aeruginosa*. A narrowing of the molecular weight distribution towards higher molecular weight and a significant increase of molecular weight of oxo-PE were observed after six weeks (42 days). The interpretation of these results was that *P. aeruginosa* was only able to access the low molecular weight oxidation products of the pro-oxidant mediated abiotic degradation of PE (*M*_w_ < 5000 Da) and that biodegradation was only limited to the surface of the polymer and did not proceed any further.

These observations by Reddy and co-workers [28] suggest that residues of the polymer above *M*_w_ = 5000 Da might be resistant to biodegradation and might accumulate in soil. Significant differences in molecular weight before and after incubation with microorganisms were observed in studies by Fontanella *et al.* [24], Koutny *et al.* [21] and Abrusci *et al.* [27]. Chiellini *et al.* [19] and Kawai *et al.* [33] have found that fragments with a *M*_w_ around 1500 Da were more rapidly biodegraded. While Jakubowzic [23] found that only oligomers with *M*_w_ below 5000 Da can be bioassimilated by microorganisms. It is not clear if these results are compatible with the PAS 9017 : 2020, which requires that *M*_n_ needs to be below 5000 Da with a *M*_w_ mass loss higher than 90% after abiotic degradation.

More recently, Eyheraguibel *et al*. [29] studied the molecular composition of oligomers generated after abiotic oxidation of an oxo-HDPE, and after incubation with *R. rhodococcus*, by a combination of ^1^H NMR spectroscopy and mass spectrometry. The authors suggested that the extent of oxidation as well as the length of the carbon chain play an important role in the biodegradation step. In other words, it was found that longer oxidized molecules disappeared more rapidly than smaller ones as a result of the action of extracellular enzymes secreted by *R. rhodochrous*. This is in contrast with previous findings where it was thought that short chain molecules were firstly taken up by cells and that only oligomers with a molecular weight lower than 5000 Da were available for biodegradation. However, as Eyheraguibel *et al.* [29] note, in some of the previous studies [21,22,28,32] oligomer analysis was performed by size exclusion chromatography, and higher molecular weight oligomers may not be extracted when using water or acetone. Overall, this study gives a new insight into the extracellular mechanisms, but does not give information about the complete biodegradation of oxo-HDPE. Moreover, the fate of the aliphatic oligomers with a low oxidation level was not highlighted here.

Biodegradation of PAC plastics have been studied both in complex media like soil, river and sea water, and under controlled experimental conditions, e.g. with identified microbial strains. Experiments performed using microbial consortia from soil, compost or fresh/sea water are more representative of real environmental conditions, but they are also more complex, less reproducible and harder to compare, due to the range of organisms present with, for example, different growth profiles. Instead, cultures with single bacteria have the advantage that the abiotic and biotic factors responsible for the degradation of oxo-containing polyolefins can be better distinguished. Nevertheless, in the real environment, plastic is not the only carbon source for microorganisms, and the rate of biodegradation could be overestimated in laboratory tests. The level of mineralization is measured by the amount of CO_2_ produced by microorganisms during bioassimilation by a respirometric method according to international standards. However, this method could lead to an underestimation of the biodegradation levels if the production of new biomass is significant [23] and does not take into account changes in enzymatic activity [7]. Alternatively, some studies have correlated the degree of bioassimilation with the metabolic activity of microorganisms by adenosine triphosphate (ATP) and adenosine diphosphate (ADP) measurements [21,24,25,29]. For example, in the study by Fontanella *et al.* [24], ATP/ADP measurements were used to obtain information on the metabolic activity of *R. rhodochrous* upon incubation with the different oxo-PE films examined. They found that the metabolic activity was not influenced by a lack of oxidized fractions (*M*_w_ < 5000 Da), which were produced in all cases upon ageing of the oxo-PE films, but rather by the presence of high concentrations of cobalt stearate in one of the polymer blend formulations. The same methodology was later adopted in the French standard AFNOR AC TSI 808 to assess the biodegradability of PAC plastics. However, the ATP/ADP method has been criticized for not giving an indication of the absolute levels of biodegradation [4].

Recently Rose *et al.* [30] investigated the microbial degradation of several plastic materials by monitoring CO_2_ production by gas chromatography (GC). This method was employed to overcome the difficulties associated with the use of biochemical assays (e.g. ATP/ADP assay) that require removal of the biofilm from the plastic surface, which could cause potential inaccuracies during quantification analysis. This study, along with that by Eyheraguibel discussed above, are part of the ANR OXOMAR Project DS0103 *Abiotic and biotic degradation and toxicity of oxo-biodegradable plastics in the marine waters*. Although the aim of the project was to study the biodegradability of ‘oxo-biodegradable plastics' in the marine environment, the LDPE film containing the pro-oxidant d2w by Symphony Technology was only tested against the soil bacterium *R. rhodochrous*. Moreover, only a conventional LDPE film and a bioplastic, defined as a ‘compostable duplex laminate’, were tested for biodegradability by the marine bacterium *Alcanivorax borkumens*.

Further studies within the OXOMAR project were carried out by Dussud *et al.* [35] and Odobel *et al.* [36] on the colonization of several plastics by marine microorganisms, including PAC plastics. Evidence of bacterial adhesion and biofilm maturation were found in both studies. In the study by Odobel *et al.*, unaged oxo-PE and UV-aged oxo-PE for 48 h were visually intact after 3 days and seven months of immersion in seawater. UV-aged oxo-PE aged for 144 h was ‘very breakable’ after 3 days but it did not completely biodegrade after seven months of immersion in seawater, as fragments could still be visually observed.

### 3.2. Environmental conditions and timescales for biodegradability of pro-oxidant additive containing plastics in the unmanaged natural environment

A few studies have tried to find a correlation between laboratory tests and outdoor exposure. In 2019 Vazquez *et al.* [37] studied LDPE, HDPE and PP blended with 1% and 2% by weight of pro-oxidant under accelerated laboratory weathering and natural outdoor exposure for 4 years (humidity-free conditions; room temperature). All films showed significant degradation after both abiotic processes and a reduction in molecular weight. However, there were some methodological discrepancies in this paper. Firstly, the initial molecular weights of the PE films (with and without pro-oxidants) before artificial or outdoor weathering were not included in the paper. Secondly, the molecular weights of the UV-aged samples were significantly lower than those from the outdoor exposure experiment, making comparisons of the biodegradability difficult to validate.

Recently, two studies were published in 2021 by the same authors [38,39] to examine the correlation between artificial laboratory UV-accelerated weathering and the outdoor exposure of PAC plastics according to the new PAS 9017 : 2020. To the best of our knowledge these are the only two studies reported in the literature that use the pass/fail criteria of the PAS to assess the abiotic degradation of PAC plastic films under laboratory UV-accelerated weathering and outdoor exposure.

Both articles compared the abiotic degradation by artificial laboratory UV-accelerated weathering and outdoor weathering of two different polyethylene films, with or without the addition of a PAC additive (MB) dosed at 2% w/w. According to the cited patent [40] the samples under study contained, amongst other additives, transition metal salts/complexes which are the catalysts used to accelerate the rate of photo-/thermal degradation of polyolefins. In the first study [38], a 87.5 µm thick polyethylene film consisting of a 75% : 25% w/w LDPE/LLDPE blend with or without PAC MB additive was tested. Artificial laboratory weathering was carried out according to BS PAS 9017 : 2020 for 14 days. Outdoor weathering was performed at the Q Lab Corporation testing site in Miami, Florida, according to ASTM D1435, for 90 days. In the second study [39], a film consisting of 52% LLDPE, 40% mLLDPE, 5% VLLDPE and 3% Green MB by weight was used as a control. For films containing 2% of PAC MB additive the LLDPE component was reduced to 50%. The thickness of all the examined films was kept constant at 17 µm. Artificial weathering was performed according to BS PAS 9017 : 2020. Outdoor weathering was carried out in Miami (Q-Lab Weathering Research Service) and Sanary-sur-Mer, France (Atlas Material Testing Technology B.V.) for 90 days and 120 days, respectively. Control samples were not reported for the 17 µm films in Miami outdoor weathering tests.

The degradation of all films was assessed by following the CI by ATR-FT-IR and the molecular weight reduction (*M*_n_, *M*_w_, *M*_z_) by high-temperature GPC over time, as required by the PAS 9017. The 17 µm films in the second study were also tested by a drop point test, a softening/melting temperature measurement, to assess the formation of polyethylene waxes upon weathering. Runtime fractions (time_i_/total time) were used in the two studies to compare the results from laboratory accelerated weathering and outdoor exposure done at different times (days).

In general, a good correlation was found between the samples tested under laboratory and controlled outdoor exposure. The films containing the PAC MB additive showed a much higher degree of degradation compared with the control samples. A drop melting point test was used to determine whether the degradation of the films led to the formation of waxes, according to ASTM D3954-15 [41]. ASTM D1986-14 gives a definition for polyethylene waxes which is also adopted by the authors: ‘Polymerized ethylene with a molecular weight 2,000 to 10,000 g/mol and a density of 0.9 to 1.0 g/cm^3^. These polymers may be oxidised or polymerized but should have a melting point below 140°C [42]. All films containing the additive met this requirement after weathering, whereas the films without the additive showed a drop point above 140°C. This is consistent with the presence of the additive accelerating film degradation and conversion into a wax [39]. This is evidence that the PAS 9017 : 2020 accelerated test standard does predict aspects of the degradation of PAC plastics in the South of France and Florida.

The critiques of these studies focus on some discrepancies regarding the molecular weight determination, CI measurement*s*. These are discussed below:

1. Molecular weight determination:
1.a.) In the second study [39], the control sample tested in the South of France showed a 67% *M*_w_ loss, which exceeded the 27% *M*_w_ reduction observed for the same control sample in laboratory weathering test using PAS 9017; thus the correlation between artificial and outdoor weathering is less clear in this case.
1.b) The control sample for the Florida test was not included in the second study [39], so a comparison/correlation between samples cannot be made.
1.c) For the purpose of PAS 9017 more than one replicate is required. However, in both studies the authors only reported one molecular weight measurement per sample.
1.d) In both studies, all films, with and without the pro-oxidant additives, were produced by an extrusion process. Molecular weight analysis shows that the *M*_n_, *M*_w_ and *M*_z_ values after extrusion but before any artificial or outdoor weathering (*t* = 0 days) for the control samples differ from that of the samples containing PAC MB additive. The data are summarized in the table 5. A conclusion from this data is that, during film extrusion, the presence of the additive reduces the final molecular weight of the polymer in the film. This was also previously observed by Vazquez [37]. The reduction in molecular weight is around 35% for the 87.5 µm films and 43% for the 17 µm films, when compared with their respective control samples. At the same time, the increased PDI values for the film samples containing the additive indicates a much wider spread of molecular weight distribution. Since the control samples (without additive) and the samples containing the additive are different in terms of polymer structures/properties before weathering it is difficult to make direct comparisons.
2. Carbonyl index measurements:
CI measures the extent of oxidation of polyolefins as a result of the weathering process. This value should be greater than 1.0 after artificial or outdoor weathering, as specified in PAS 9017, for a film sample to be qualified for soil-biodegradability. In the second study [39], the CI measured for the 17 µm films containing the PAC additive after 120 days of outdoor exposure in the South of France was 0.62, significantly below the 1.0 threshold required by PAS 9017. However, the same film met the required molecular weight criteria of PAS 9017 after 120 days. In contrast, when this film was exposed to laboratory artificial weathering, the molecular weight and CI criteria were met after roughly the same exposure time (table 6). This mismatch could imply that different oxidative pathways might be involved in laboratory and outdoor weathering of the same film sample. Further studies are required to address the validity of correlations between the two test methods.

**Table 5.** Molecular weights of films after extrusion but before artificial or outdoor weathering (*t* = 0 days).

	*M*_n_ (Da)	*M*_w_ (Da)	*M*_z_ (Da)	PDI^a^
article 1 [38]	87.5 µm films (75% LDPE, 25% LLDPE)			
control without PAC additive	32 280	219 680	1 263 734	6.8
sample with PAC additive	20 963	239 603	845 366	11.4
article 2 [39]	17 µm films (52% LLDPE, 40% mL LDPE, 5% VLLDPE, 3% green MB)			
control without PAC additive	37 616	110 118	251 393	2.9
sample with PAC additive	21 554	151 588	495 400	7.0

^a^PDI = polydispersity index = *M*_w_/*M*_n._

**Table 6.** Table showing the CI, *M*_n_, *M*_w_ and *M*_z_ values for the sample containing PACMB additive after laboratory accelerated and outdoor exposure from article 2 [39].

17 µm films (52% LLDPE, 40% mLLDPE, 5% VLLDPE, 3% green MB, 2% PAC MB additive)					
time (days)	runtime fraction	CI	*M*_n_ (Da)	*M*_w_ (Da)	*M*_z_ (Da)
laboratory artificial weathering					
0	0	0.09	21 554	151 588	459 400
14	1	1.60	1956	5397	11 929
outdoor weathering in the South of France					
0	0	0.09	21554	151 588	459 400
120	1	0.62	2119	9532	25 834

### 3.3. Summary and conclusions

Two studies published in 2021 [38,39] provide evidence for the abiotic degradability of the films containing the additive under the conditions specified in the PAS. However, further investigation is needed for the purpose of the PAS 9017 : 2020 to understand if the endpoint reached by the films after weathering, defined in the two studies by the formation of waxes, does confer soil biodegradability properties. Most of the laboratory tests of PAC plastics studied show biodegradability values in the range 5–60%. Only Jakubowicz's work [23] showed a biodegradability that was higher than 90%, which was reached in simulated soil after 733 days. The different levels of biodegradation found in most studies were correlated to the different protocols and experimental conditions (UV irradiance, temperature, length of exposure, humidity) adopted to investigate the abiotic degradation of PAC plastics, which is a critical step as it determines the rate of biodegradation by microorganisms. However, these studies failed to provide a precise timescale for the abiotic degradation of PAC plastics in the unmanaged natural environment. A clear correlation between laboratory studies and the conditions natural outdoor weathering is lacking. Moreover, limitations were found when assessing the biodegradability of PAC plastics under controlled laboratory conditions, due to a lack of standardization of the types and concentration of microorganisms, and limitation of the current test methods employed.

Both studies from 2021 [38,39] gave a timescale and evidence of correlation between the abiotic degradation of film samples upon accelerated laboratory weathering and outdoor exposure in South of France and/or Florida according to the criteria of PAS 9017 : 2020. However, in climates such as the UK, central and northern Europe with their lower temperatures, lower sunshine hours and UV intensity, degradation under PAS guidelines might take longer than the four months in the South of France or 90 days in Florida. Outdoor weathering tests in climatic conditions more similar to the UK, central and northern Europe need to be carried out to assess realistic timescales in climates with less favourable conditions for the abiotic degradation of polyolefins.

Thus in answering the question posed in this section ‘Is there evidence that correlates laboratory weathering tests of PAC plastics to their behaviour in the unmanaged natural environment?’, we conclude:

(1) There is evidence that a new standard PAS 9017 : 2020 is relevant to predicting the timescale for abiotic stage of PAC plastic degradation in hot dry climates.
(2) There is little data to substantiate the claim that PAC plastics fully biodegrade after the abiotic degradation stage.
(3) There is not sufficient evidence at present to equate the formation of waxes after abiotic degradation with the claim of biodegradability.
(4) There is no data at present to substantiate the claim that the PAS 9017 : 2020 predicts the timescale for abiotic degradation of PAC plastics in cool or wet climatic regions such as the UK or under less ideal conditions (soil burial, surface soiling etc.).

## 4. What are the findings from real field-test studies?

There are few studies assessing the biodegradation of PAC plastics in real-world conditions. The key publications are summarized in table 7. Agricultural mulch films containing pro-oxidants will also be highlighted in the discussion below. Mulch films are used to protect and enhance cultivation yields, reducing the use of water and pesticides, which results in better food quality and safety [49]. PAC mulch films have been proposed as a solution to reduce the costs associated with the collection and disposal of conventional films. They are designed to be left on site and mixed with soil to ultimately biodegrade.

**Table 7.** Key peer-reviewed publications for field trial studies.

key peer-reviewed publications	reference	material tested	laboratory controlled study	field trial
Feuilloley 2005	[43]	oxo-PE mulch films	no pre-ageing, 15% biodegradability after 200 days	Soil burial for 1 year, after 2 years of cultivation; 90% degradability by visual inspection; evidence of micro-fragments (5–70 mm) and cross-linking.
Briassoulis 2014	[44]	oxo-LLDPPE mulch films	no	soil burial (8.5 years); evidence of micro-fragments (5–70 mm) and cross-linking.
Briassoulis 2015	[45]	oxo-LLDPPE mulch films (same as previous work)	pre-aged (50°C, 800 h) + exposure to high-intensity UV light (decades of ageing in real environment)	soil burial (7 years); evidence of micro-fragments (5–70 mm) and cross-linking.
Gauthier 2015	[46]	oxo-PE mulch films	UV-laboratory-accelerated weathering exposure to different soil from field trial sites; different concentration of organic matter in soil influences the rate of degradation	surface soiling in three different climatic Australian locations; different time to embrittlement found for same samples in different locations
O'Brien 2010	[47]	conventional PE carrier bag, oxo-PE carrier bags (TDPA by EPI Inc), one compostable carrier bag	no	submerged below sea surface in Plymouth UK; less than 2% of surface area loss for oxo-PE after 16 weeks; bags became highly fouled; compostable bags completely disappeared
Napper 2019	[48]	one conventional PE, one compostable, one biodegradable and two oxo-PE carrier bag	controls kept in the dark at room temperature	open-air; soil burial; submerged in marine environment for 3 years in Plymouth UK

Feuilloley *et al.* [43] investigated the biodegradability of three commercial mulch films sold as soil biodegradable, Material A (starch based), Material B (aliphatic/aromatic polyester) and Material C (PE) a PAC plastic containing pro-oxidants, according to 10 standardized test methods. All experiments on PE were performed without UV or thermal treatment. The rationale behind this was that 30–50% of agricultural films are buried in the soil during film casting and will not be exposed to strong UV light or strong temperature that are often employed in laboratory studies. The biodegradation of the three films was tested in different conditions such as compost, anaerobic, enzyme test, soil test etc. Overall, Material A proved to be the most biodegradable film, followed by Material B and Material C, regardless of the test conditions used. In particular, for the experiments carried out in soil under controlled laboratory conditions (under test standard DIN 53 739 [50]), Material C reached a plateau after 200 days and no more than 15% biodegradability was observed overall. Films were also tested in agricultural soil where they had been used for mulching for two years and then buried in soil for 1 year. In this case, Material C showed 90% degradation by visual inspection after 11 months.

Briassoulis *et al.* [44] studied the degradation of PAC LLDPE mulching films after their burial in soil under real field conditions but for a total duration of 8.5 years following their use in watermelon cultivation. At the end of the cultivation period (eight months) but before soil burial, LLDPE films exhibited a severe reduction of their mechanical properties by 50%, an increase in CI and crystallinity. After the long soil burial period of 8.5 years, PAC LLDPE mulching films remained intact. Spectroscopic analysis revealed that the CI decreased to a value close to zero. Thermal analysis measurements showed a decrease of up to 32% in the degree of crystallinity.

In a later work by Briassoulis *et al.* [45] the same PAC LLDPE mulching film used for the watermelon cultivation was artificially aged either by thermal oxidation (50°C, 800 h) or by exposure to high intensity UV-A and UV-B radiation. The authors suggested that these conditions should correspond to ‘decades’ of ageing in the real environment. A LLDPE film without pro-oxidant was used as a control. After abiotic treatment, thermally and photo-aged PAC LLDPE were buried in the soil for 7 years. Crystallinity and the CI followed the same trend observed in the previous study showing an increase during the pre-ageing phase and a decrease during the soil burial test. These findings are also in agreement with those from Feuilloley's work [43] and suggest that during photo-ageing and in conditions where there is little oxygen, more cross-linking reactions occur rather than chain breaking in the polymer. Retrieved virgin LLDPE samples were completely intact after 7 years, followed by a higher fragmentation of thermally pre-aged samples.

In another study, Gauthier *et al.* [46] investigated the effect of different types of soil on the degradation of PAC PE films. These films were exposed to natural outdoor weathering conditions above ground during the Australian mid-spring (2009) across three trials sites which differed in the type of climate and soil, the latter distinguished in terms of organic matter (OM). Time to embrittlement was evaluated as ‘the ageing time elapsed until the film fractured multi-directionally when a small stress was manually applied normal to the film plane’. A complete description of the soil characteristics is reported in the supporting information of this paper. To confirm that a site-dependent factor (other than UV, temperature and rainfall) was impacting the rate of photo-oxidation, soil samples were taken from the three different trial sites and UV pre-oxidized PE films were exposed over these soils under controlled laboratory conditions. Other samples included in the test were: control samples without the pro-oxidants; samples tested in the air; sample tested in water, as this is one of the main components of soil that can have an important effect on the photo-oxidation of the sample; samples with either humic acid (HA) or fulvic acid (FA), which are other important components of the soil organic matter. The film with the pro-oxidant showed shorter times to embrittlement than the virgin polymer under all conditions. All samples exposed to air showed photo-resistance and the time to embrittlement was the highest. The effect of soil was evident by a decrease in the time of embrittlement with the increase of OM. The authors attributed this effect to a synergic action of water in the presence of HA and FA in the organic matter within the soil.

Fewer studies have investigated the levels of biodegradability of PACs in the aquatic environment due to the high complexity of this system. In a 2010 study by O'Brien & Thompson [47], one conventional PE carrier bag, two PAC PE carrier bags, and one compostable bag were submerged below 0.6 m from the sea surface in Plymouth UK. After 40 weeks the tensile strength of all materials was reduced significantly with the greatest effect for the compostable bag, followed by the PAC plastic carrier bags and the conventional bag. Less than 2% of surface area was lost for the conventional PE and PAC plastic carrier bags. The compostable bag showed progressive surface loss up to 16 weeks, after which the sample completely disappeared. Labelling from manufacturer of the PAC PE carrier bags stated that the degradation process should start after 18 months and complete degradation would take 3 years, thus the test period was not appropriate to test the claims of this PAC material.

In another study five different types of plastic carrier bags were studied by Napper & Thompson [48]: two types of PAC plastic bags, one biodegradable plastic bag, one compostable plastic bag and a conventional HDPE bag. Deterioration studies were carried out over a period of 3 years across three natural environments: open-air, buried in soil and submersed in the marine environment, and under controlled laboratory conditions (room temperature, in the dark). All field trials were carried out in Plymouth, UK. Three sampling dates were used: 9 months, 18 months, 27 months. Statistical analysis was used in this study. Deterioration was considered in terms of the visible loss of surface area, tensile stress, surface texture and chemical structure. Visual observations are summarized in table 8.

**Table 8.** Visual observation for oxobio1, oxobio2, biodegradable, compostable and conventional carrier bags in different environments (laboratory, open-air, soil, marine environment) over two periods, 0–9 months and 9–27 months.

	oxobio1	oxobio2	biodegradable	compostable	conventional
0–9 months					
laboratory control	no surface area loss				
open-air	—	—	—	—	—
soil	no surface area loss				
marine env.	biofilm formation	biofilm formation	biofilm formation	no longer visible	biofilm formation
9–27 months					
laboratory control	no surface area loss				
open-air	too brittle to be tested				
	pieces were in the microplastics size range (less than 5 mm)				
soil	no surface area loss				
marine env.	biofilm formation	biofilm formation	biofilm formation	no longer visible	biofilm formation

Biofilm formation was observed for all bags, including conventional bags. All bags in the open-air environment fragmented to small pieces in the microplastics size range (less than 5 mm). Loss of mechanical properties was found for all bags and it was more significant for the samples exposed to outdoor sunlight. This is not surprising, as the amount of UV exposure and oxygen would be less for samples buried in soil, landfill or submerged in the marine environment. Compostable bags completely disappeared from the marine environment and in agreement with the study of O'Brien & Thompson [47]. The PAC plastic, biodegradable and conventional bags retrieved from soil and marine environment after 3 years were still functional in holding typical groceries (2.25 kg) while compostable bags were intact but unable to hold any weight without tearing.

### 4.1. Summary and conclusion

Field trials are useful to study the degradation of PAC plastics under real environmental conditions and provide a rough prediction on the degradation rate of a plastic material in a specific environment where it may end up as litter. However, due to the high complexity of these systems, there is a lack of standardization of test methods to assess the biodegradability of PAC materials in the natural environment. The results of field studies show that that PAC plastics can remain in the environment for years. What is evident from both laboratory and field studies is that the abiotic degradation is a crucial step for biodegradation to take place. PAC mulch films did degrade under certain conditions, forming biofilms on their surface, but the rate of biodegradation was slow. However, the timeframe needed to achieve the right level of oxidation during the abiotic step is not clear as it not only depends on the polymer structure, type and ratio of pro-oxidants to UV stabilizers, but also on the environmental conditions (pH, temperature, types of microorganisms, UV light exposure etc.), which can vary significantly across different environments and even within the same environment.

Thus in answering the question posed in this section ‘What are the findings from real field-test studies?’, we conclude that currently there is no reliable data to substantiate the claim that the PAC reliably biodegrade in real conditions across as range of environments and climates.

## 5. Are microplastics formed during the degradation of pro-oxidant additive containing plastics?

The biodegradation of plastics usually results in the production of microplastics which are resident in the environment for a period of time. Assessing the length of that time is crucial to understanding the environmental impact of PAC plastics. Few studies have been carried out to assess microplastics formation arising from PAC plastics. Feuilloley *et al.* [43] have investigated the biodegradability of three commercial mulch films sold as soil biodegradable including a PAC plastic. Films were tested in agricultural soil where they had been used for mulching for 2 years and then buried in soil for 1 year. The PAC plastic material showed 90% degradation by visual inspection after 11 months, producing micro-fragments (5–70 µm). These fragments were recovered and observed by optical polarized microscopy. Briassoulis *et al.* [45] also found that invisible (to the eye) micro-fragments smaller than 1 mm were observed by optical microscopy for the oxo- LLDPE mulch film after 7 years of soil burial.

Yang *et al.* [51] also investigated the possible formation of microplastics in soil for different types of mulch films: two conventional PE (white and black), one PAC PE and one ‘biofilm’ (70% polybutylene adipate terephthalate, PBAT). Samples were buried in soil collected from a cultivation site in Beijing, China and exposed to a xenon-arc lamp (ISO 4892-2) and collected at different exposure times, 28, 42, 56 and 70 days. Microplastics released from the deteriorated mulch films into soil samples were extracted, stained with Nile red and analysed by a fluorescent stereomicroscope. Microplastics were detected after 28 days of UV irradiation and the amount of detectable microplastics increased with longer photo-oxidation periods. On the 70th day of UV ageing, the ‘biofilm’ had the highest rate of microplastics with 475 particles cm^−2^ followed by PAC PE with 265 particles cm^−2^, PE white mulch films with 163 particles cm^−2^ and PE black mulch films with 147 particles cm^−2^.

The most recent studies of PAC plastics [38,39] showed that the endpoint of the degradation/weathering process resulted in the formation of waxes, and the authors indicated that microplastics are not formed during the degradation of the film containing the PAC additive. By contrast, the films without the additive showed a slower degradation and the authors speculated that microplastics might therefore form during the erosion of the polymer. However, analyses were not reported to confirm this. It has been noted that because the morphology and structure of microplastics is different from that of unaged plastics or larger debris, their behaviour and bioavailability may be different from the original material, e.g. different biofilm adhesion, adsorption and diffusion of organic pollutants and trace metals (depending on the crystalline/amorphous ratio) [52].

A proportional increase in crystallinity was observed for all samples studies by Yang *et al.* [51] with UV exposure times (table 9). These data highlight that the rate of abiotic degradation of the polymer increases with longer exposure times and thus it would make the polymer more accessible to microorganisms. As the amorphous phase of the polymer is ‘consumed’, the crystallinity will increase and so further degradation by both abiotic and biotic factors is likely to decrease. Eventually, this process may leave microplastics and/or nanoplastics with high crystallinity. Halle *et al.* [52] found that even if microplastics in seawater are constantly eroded, no significant chemical changes (e.g. CI) were detected. Conversely, their crystallinity increased with decreasing particle size as amorphous regions were degraded more rapidly than the crystalline regions.

**Table 9.** Crystallinity percentage (%) for PE white, PE black, oxo-PE and biofilm at 0 days and 70 days. Reproduced from Yang *et al.* [51].

ageing time (days)	crystallinity (%)			
	PE white	PE black	oxo-PE	biofilm
0	30.06	30.05	26.79	58.94
70	33.56	30.26	32.84	73.77

### 5.1. Summary and conclusion

To date, there are few studies that have been performed to study microplastic formation in PAC plastics. Of those that do exist there is evidence that microplastics are formed. The exact mechanisms of microplastic formation are thought to relate to the transformation of their physical and chemical properties through the process of cross-linking and increased crystallinity.

We conclude that microplastics form during the biodegradation of all plastics in the open environment, PAC plastics are no exception. More work needs to be done to assess the formation and lifetime of microplastics created from PAC plastics.

## 6. Are there any ecotoxicity issues relating to the pro-oxidant additive containing plastic additives?

According to the patents pertaining to PAC plastics [40], their pro-oxidant ingredients typically include transition metals which will end up in the environment. Leaching of potential toxic chemicals resulting from fragmentation of the PACs upon abiotic treatment has received little attention. Levels of Co stearate found in Fontanella *et al*.'s studies from 2011 and 2013 [24,25] were above (*ca* 10-fold) those permitted in UK rural soils [53]. High concentrations of Co in soil have shown detrimental effects on plant growth and metabolic functions [54].

Al-Salem *et al.* [55] found that one of the PAC plastic bags studied had a high content of heavy metals such as lead (Pb; 3584 ppm) whose values should not exceed the soil guideline values (SGV) of 80 ppm in UK soils (allotments land-use) [56]. Pb is a neurotoxin, potential carcinogen and could cause infertility [57]. Another threat is that Pb could persist in the environment and have adverse effects on seed germination [58].

One report evaluated the ecotoxicological effect of PAC plastics on the germination or development of tomato plants, and it did not show any adverse effect [59]. In other work by Sable *et al.* [60] PP photo-aged film samples containing Co stearate as the pro-oxidant were tested against mung bean and wheat plants and earthworms. None of these films was found to be toxic against earthworms, and the seedlings in the growth medium showed that the average plant growth levels were the same. Schiavo *et al.* [61] carried out a detailed study on the potential toxicity of leachates from 1.6 mm fragments of different PAC plastics. Leachates extracted were used in acute chronic and genotoxic tests on bacteria (*Vibro fisheri*), crustaceans (*Daphnia magna*) and plants (*Sorghum. saccharatum, Sinapis alba* and *Lepidium sativum*). Elemental analysis showed that Ca and Mg were present in high amounts in all polymers but only Cd was at a concentration of 9.94 µg l^−1^ above the permitted limits (5 µg l^−1^) for drinking water and spring water in England [62,63]. Analysis of organic compounds released from the polymer leachates showed similar levels for all polymers under study. Different isocyanate derivatives were also consistently found in the water leachates, but complete determination of all organic compounds was not possible. The highest toxicity effect was observed for *Daphnia magna*, followed by *Vibro fisheri* and plants. In a previous study by Schiavo *et al.* [64], leachates from virgin PE, PP and PS were tested against *D. magna* and a lower toxicity was observed in this case compared with those observed for the pro-oxidant containing polymers. The authors suggested that the presence of pro-oxidants accelerated the release of metals and potentially other toxic compounds, increasing adverse effects compared with the respective virgin polymers.

### 6.1. Summary and conclusion

There are few systematic studies of the ecotoxicity of PAC plastics. Those that do exist show that metal additives from the PAC plastics do end up in the soils and in water, sometimes in high proportions that exceed recommended or permitted concentrations. Ecotoxicity studies on plant germination or earthworms have not shown any harmful effects. More ecotoxicity studies are needed to assess the possible effect of transition metals accumulation in the environment.

We conclude there is not reliable data to date to reliably assess the ecotoxicity of PAC plastics in the many different environments of the open unmanaged environment. Ecotoxicity studies are needed to assess the possible effect of transition metal accumulation on the environment.

## 7. Implications for policymakers

There are places in the world, and particular applications (e.g. mulch films), where the waste strategy is not to collect plastics but to let them biodegrade in the open unmanaged environment, ideally causing no harm. In this paper we have reviewed the evidence to understand whether PAC plastics can be part of such a solution. We have addressed outstanding questions regarding the biodegradability and environmental impact of PAC plastics in the unmanaged natural environment.

In §3 we reviewed the evidence for the biodegradation of PAC plastics, which showed that there is no data as yet that they biodegrade effectively in the unmanaged natural environment. There is evidence that PAC plastics undergo accelerated abiotic degradation (the first stage of biodegradation) and this evidence shows a correlation between accelerated laboratory weathering and outdoor exposure in South of France and/or Florida according to the criteria of PAS 9017 : 2020. However, when biodegradation is measured directly in laboratory tests, the values measured are in the range 5–60% and would not pass the criteria for biodegradability set in the test standard PAS 9017 : 2020. What is evident from both laboratory and field studies is that the abiotic degradation is a crucial step for biodegradation to take place. However, the timeframe needed to achieve the right level of oxidation during the abiotic step is not clear. This timeframe not only depends on the polymer structure, type and ratio of pro-oxidants to UV stabilizers but also on the environmental conditions (pH, temperature, types of microorganisms, UV light exposure etc.), which can vary significantly across different environments and even within the same environment. Thus it is very hard for a single material formulation to suit all conditions, all weathers, all soil types and all geographies. This is possibly why the existing evidence reviewed in §4 on field trials showed that PAC plastics do not reliably biodegrade in real-world settings.

The other issue raised by our review is the choice of testing sites and how this related to the generality of claims about the performance of PAC plastics. Recently, work showed that 60% of home-compostable plastics did not fully biodegrade in UK home composting conditions [65]. The research showed that the biodegradable plastics experienced very different conditions in the home setting, and yet a single type of laboratory test was being used to certify composability. As a result, although manufacturers claimed ‘100% biodegradability’ because their materials passed the laboratory test, the reality was that most of the plastics did not biodegrade in real-world settings [65]. The abiotic degradation step of the PAS 9017 : 2020 laboratory test has been correlated with outdoor exposure from the South of France and Florida; however, we know that the rates of abiotic degradation will be lower in cooler and wetter climates with lower levels of sunshine, such as those in the UK, North America and northern Europe. Thus it is appropriate to express caution about the ability of the current version PAS 9017 : 2020 to be predictive of the biodegradability of PAC plastics in the open unmanaged environment in a wide variety of climates. We suggest that PAS guidelines should include field testing in realistic conditions in every country or territory where PAC plastics are planned to be deployed.

When PAC plastics degrade in the environment, we do not really know how many microplastics are formed or how they persist in the environment. This is also true of other plastics. In the case of PAC plastics an important piece of evidence to understand is the mobility of PAC microplastics once formed. This is because these microplastics may be buried in soil, blown in the wind, or become part of a body of water. In each case there is likely to be a different fate for that plastic because its environmental conditions have changed, as will their rate of biodegradation. For instance, we know that environmental conditions such as UV exposure and heat determine the abiotic step of degradation, so if a microplastic becomes buried in a soil its rate of degradation will be much reduced. Also cross-linking is more likely to happen in soil, landfill and the marine environment, due to the reduced amounts of oxygen available in these systems; this leads to lower levels of degradation.

We found that there is little data assessing the risks of ecotoxicity from the PAC plastic additives entering the environment. In the case of mulch films such evidence is vital to assessing the impact on crops and soil health. In the case of other applications of PAC plastic, their ecotoxicity is likely to be related to where they end up in the environment and their concentrations. Until the risks of ecotoxicity and microplastics of PAC plastics are better known, the impact on the unmanaged natural environment is difficult to assess. We, however, advise caution, since the impact of conventional plastics on the environment and ecosystem is already very large [66], and there is no evidence to show that PAC plastics are less harmful than conventional plastics.

## 8. Conclusion

Overall we conclude there are several issues that need to be resolved for any country or territory wanting to employ PAC plastics as part as their waste management strategy.

The conclusions can be summarized as follows:

— There is evidence that PAC plastics abiotically degrade in the open unmanaged environment in hot dry climates characterized by the South of France and Florida, but the timescales for complete biodegradation in the open unmanaged environment of these climates are as yet unknown.
— The timescales for abiotic degradation and biodegradation in other more temperate climates such as the UK are as yet unknown.
— The degree of microplastic persistence in the environment and any toxicity to microorganisms are as yet unknown.

## Data Availability

This article has no additional data.
